# Mutations in *embB*406 Are Associated with Low-Level Ethambutol Resistance in Canadian *Mycobacterium tuberculosis* Isolates

**DOI:** 10.3390/antibiotics13070624

**Published:** 2024-07-04

**Authors:** Morgan Hiebert, Meenu K. Sharma, Melissa Rabb, Lisa Karlowsky, Kiana Bergman, Hafid Soualhine

**Affiliations:** 1 National Reference Centre for Mycobacteriology, National Microbiology Laboratory, Public Health Agency of Canada, Winnipeg, MB R3E 3R2, Canada; morgan.hiebert@phac-aspc.gc.ca (M.H.); meenu.sharma@phac-aspc.gc.ca (M.K.S.); melissa.rabb@phac-aspc.gc.ca (M.R.); lisa.karlowsky@phac-aspc.gc.ca (L.K.); kiana.bergman@phac-aspc.gc.ca (K.B.); 2 Department of Medical Microbiology and Infectious Diseases, University of Manitoba, Winnipeg, MB R3E 0J9, Canada

**Keywords:** ethambutol, resistance, *embB*, *Mycobacterium tuberculosis*

## Abstract

In *Mycobacterium tuberculosis*, molecular predictions of ethambutol resistance rely primarily on the detection of mutations within *embB*. However, discordance between *embB*406 mutations and gold standard phenotypic drug sensitivity testing (DST) questions the significance of *embB*406 mutations used in molecular DST. This study tabulates *embB* mutations found in Canadian *M. tuberculosis* isolates and evaluates the impact of specific mutations on ethambutol resistance. The National Reference Centre for Mycobacteriology culture collection (n = 2796) was screened for isolates with *embB* mutations. Phenotypic DST was performed on the BACTEC™ MGIT™ 960 at ethambutol concentrations of 2–5 μg/mL. Whole genome sequencing was used for drug resistance predictions, phylogenomics and single nucleotide polymorphism analysis. Detection of resistance-associated *embB* mutations corresponded to a positive predictive value of 64.3%, negative predictive value of 99.2%, 98.7% specificity, and 73.3% sensitivity compared to phenotypic DST. Two *embB*406 mutation subtypes (Gly406Asp, Gly406Ala) were found among 16 isolates, of which 12 were sensitive at 5 µg/mL ethambutol with variable resistance between 2–4 µg/mL. A novel frameshift mutation in regulator *embR* (Gln258fs) was found in nine isolates. Mutations in *embB*406 were associated with low-level ethambutol resistance undetectable at the recommended critical concentration (5 μg/mL). These novel mutations may exacerbate variability in ethambutol resistance.

## 1. Introduction

*Mycobacterium tuberculosis* remains a priority pathogen for the World Health Organization (WHO) as the causative agent of tuberculosis and the second leading infectious cause of mortality worldwide [1]. Globally, drug-resistant *M. tuberculosis* poses a serious risk to public health, as treatment options remain limited for resistant infections [1,2,3,4]. In Canada, nearly 10% of all tuberculosis infections in 2022 were resistant to at least one first-line anti-tuberculosis drug [5].

Drug susceptibility testing (DST) for *M. tuberculosis* is performed using culture-based methods as the gold standard [6]. Specifically, mycobacterial DST relies upon the proportion method and testing at a critical concentration, defined as the minimum drug concentration required to inhibit 99% of wild-type isolates of *M. tuberculosis* but does not inhibit strains that are resistant to antimicrobial therapy in vivo [6]. These phenotypic assays are time-consuming due to the slow-growing nature of *M. tuberculosis*. Advances in molecular techniques such as PCR-based methods [7,8,9,10] and whole genome sequencing (WGS) [11,12,13] have enabled rapid detection of resistance-associated gene mutations. However, discordance between gold standard phenotypic DST and rapid molecular methods is reported for ethambutol [14,15,16].

Ethambutol is a bacteriostatic first-line anti-tuberculosis drug that targets arabinosyltransferases encoded by the *embCAB* operon, hindering the arabinogalactan biosynthetic pathway and thereby inhibiting mycobacterial cell wall synthesis [17]. Mutations in the arabinosyltransferase-encoding *embB* gene, including *embB*306, *embB*406, and *embB*497, are known to convey ethambutol resistance in *M. tuberculosis* and are employed in diagnostic pipelines for rapidly predicting resistance to ethambutol [18,19,20]. However, several studies have reported *embB*406 mutations in both ethambutol-resistant and susceptible isolates [15,16,21,22,23]. This discordance between genotypic and phenotypic DST at the current critical concentration questions the significance of *embB*406 mutations used for rapid molecular DST.

We hypothesize that *embB* mutations in codon 406 are associated with low-level ethambutol resistance which is undetectable by the current critical concentration (5 μg/mL) utilized for DST on the BACTEC™ MGIT™ 960 system. This study tabulates *embB* mutations found in Canadian *M. tuberculosis* isolates from 2002–2022, evaluates the impact of specific *embB*406 mutations on ethambutol resistance compared to the MGIT™ 960 method, and considers other *embB* mutations and novel mutations outside of *embB* which may exacerbate phenotypic variability. We found that mutations in *embB*406 were associated with low-level ethambutol resistance undetectable at the recommended critical concentration (5 μg/mL) and that novel mutations may exacerbate variability in ethambutol resistance.

## 2. Results

### 2.1. Overview of embB Mutations and Ethambutol Resistance

The National Reference Centre for Mycobacteriology (NRCM) culture collection spanning years 2002–2022 houses 2794 *M. tuberculosis* isolates which underwent both routine molecular and phenotypic DST for ethambutol. Table 1 shows the results of *embB* mutation screening and phenotypic ethambutol susceptibility testing at the critical concentration on the BACTEC™ 460TB (2.5 µg/mL) or BACTEC™ MGIT™ 960 (5.0 µg/mL) systems. In total, 3.08% (n = 86/2794) of isolates were phenotypically resistant at the critical concentration and 96.92% were phenotypically sensitive (n = 2708/2794). Further, 5.01% (n = 140/2794) isolates in the culture collection were found to harbour an *embB* mutation. Of these isolates, 45.71% (n = 64/140) were resistant and 54.29% (n = 76/140) were sensitive.

Of all the ethambutol-resistant isolates, 74.42% (n = 64/86) had a single one-nucleotide mutation in *embB*. Specifically, 60.47% (n = 52/86) of resistant isolates had a mutation in *embB*306, 5.81% (n = 5/86) had a mutation in *embB*406, and 3.49% (n = 3/86) had an *embB*497 mutation. The remaining resistant isolates had mutations in *embB*319 (n = 1), *embB*328 (n = 1), *embB*354 (n = 1), and *embB*378 (n = 1). Lastly, 25.58% (n = 22/86) of ethambutol-resistant isolates did not harbour a mutation in *embB*.

Mutations in *embB* were seen in 2.81% (n = 76/2708) of ethambutol-sensitive isolates. Specifically, 1.33% (n = 36/2708) of sensitive isolates had a mutation in *embB*378, 0.85% (n = 23/2708) had a mutation in *embB*306, 0.41% (n = 11/2708) had a mutation in *embB*406, and 0.07% (n = 2/2708) had an *embB*497 mutation. Lastly, a mutation was observed in *embB*328, *embB*226, *embB*313, *embB*333, and *embB*386 in a single isolate each. In total, 97.19% (n = 2632/2708) of ethambutol-sensitive isolates showed a wild-type genotype of the *embB* gene.

After tabulating all of the *embB* mutations found among isolates within the NRCM culture collection, the frequency of mutations in ethambutol-resistant and -sensitive isolates was used to determine the positive predictive value (PPV), specificity, and sensitivity for *embB* mutations using phenotypic DST as the gold standard. The detection of any *embB* mutation corresponded to a PPV of 45.7%, specificity of 97.2%, and sensitivity of 74.4%. An *embB* Gly406Asp mutation provided a PPV of 15.4%, specificity of 99.6%, and sensitivity of 2.3%. Finally, an *embB* Gly406Ala mutation corresponded with a PPV of 66.6%, specificity of 97.0%, and sensitivity of 2.3%. 

### 2.2. Characteristics of embB406 Mutations

**Figure 1:**
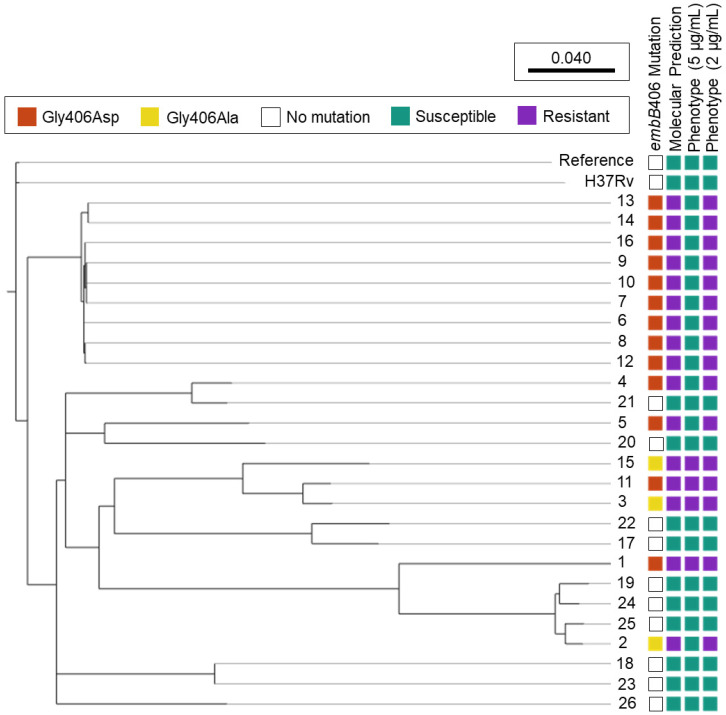
Ethambutol Susceptibility Predictions among *M. tuberculosis* Isolates Harbouring *embB*406 Mutations. Phylogenomic analysis was performed for *M. tuberculosis* study isolates 1–26 using SNVPhyl (v1.2.3) with reference *M. tuberculosis* H37Rv (NC_000962.3) and included 94.27% of all genomic positions in the core genome. The adjacent H37Rv was cultured and underwent whole genome sequencing in-house. Molecular susceptibility predictions were determined by MyKrobe Predictor (v0.10.0). Ethambutol phenotype is depicted at the critical concentration, 5 μg/mL, and a lower concentration, 2 μg/mL.

Mutations in *embB* codon 406 were the second most abundant *embB* mutation among ethambutol-resistant isolates in the culture collection, besides codon 306. Figure 1 shows the phylogenomic tree of all study isolates (n = 26) and ethambutol susceptibility based on molecular and phenotypic DST.

Of the 16 isolates with *embB*406 mutations, 13 held a Gly406Asp mutation and three exhibited a Gly406Ala mutation (Figure 1). All 16 isolates with *embB*406 mutations were predicted to be ethambutol-resistant by WGS-based molecular DST using MyKrobe Predictor. In contrast, phenotypic DST showed that just 25% (n = 4/16) isolates with an *embB*406 mutation were resistant to ethambutol at the critical concentration of 5 µg/mL. Of these four isolates, two had a Gly406Asp mutation while the other two harboured a Gly406Ala mutation. By mutation, 15.4% of all isolates with a Gly406Asp mutation (n = 2/13) and 66.6% of all isolates with a Gly406Ala mutation (n = 2/3) were resistant at 5 µg/mL. All isolates exhibited concordance between routine phenotypic DST results on the BACTEC™ 460TB system and the current BACTEC™ MGIT™ 960 system except Isolate 2, which was ethambutol-resistant on the BACTEC™ 460TB system while ethambutol-sensitive on the BACTEC™ MGIT™ 960 system. 

Conversely, 75% (n = 12/16) of isolates with an *embB* mutation were susceptible to ethambutol at 5 µg/mL, including 84.6% (n = 11/13) of isolates with a Gly406Asp mutation and 33.3% (n = 1/3) with a Gly406Ala mutation. DST below the critical concentration showed that all 12 isolates exhibited low-level ethambutol resistance (2–4 μg/mL), as described below. Finally, phenotypic DST for the 10 control pan-sensitive isolates showed that all had a wild-type *embB*406 gene locus and were sensitive to ethambutol at all tested concentrations (2–5 μg/mL).

### 2.3. embCAB Mutation Profiles and Low-Level Ethambutol Resistance among Isolates with embB406 Mutations 

**Figure 2:**
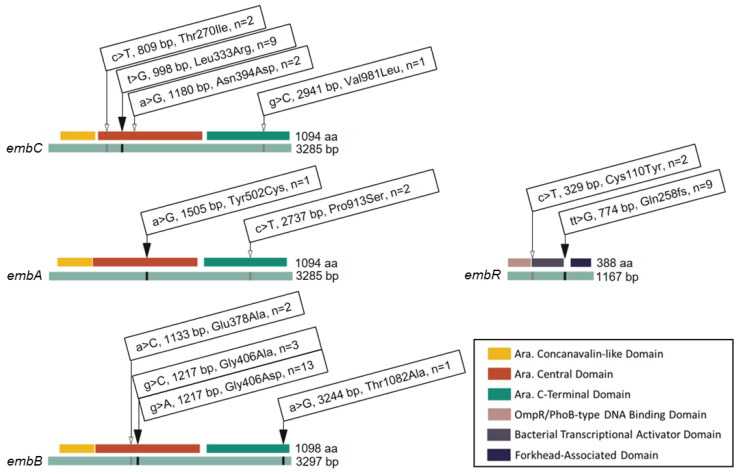
Mutations and Codon Changes Found in the *embCAB* Operon of *M. tuberculosis* Isolates. Linear gene (green) and protein domain (key) map of single nucleotide polymorphisms in the *embCAB* operon. Identity and position of base pair (bp) and amino acid (aa) substitutions are shown. n indicates the total number of ethambutol-resistant isolates harbouring a given mutation. The black arrows represent mutations found in *embB*406 mutants only. The white arrows indicate mutations found in both *embB*406 mutants and pan-susceptible control isolates.

Given the phenotypic variability of isolates with identical *embB*406 mutations, we surveyed additional mutations that may contribute to low-level ethambutol resistance (≥2 μg/mL) by WGS. Figure 2 shows all of the non-synonymous mutations that were identified within components of the *embCAB* operon, including operon regulator *embR*. In addition to the *embB*406 mutations discussed in the sections above, four mutations were found exclusively in ethambutol-resistant isolates (≥2 μg/mL). These included Leu333Arg in *embC* (n = 9 isolates), Tyr502Cys in *embA* (n = 1), Thr1082Ala in *embB* (n = 1), and a frameshift (fs) mutation in codon 258 (Gln258fs) of *embR* (n = 9). Six mutations were found in both ethambutol-resistant isolates (≥2 μg/mL) and pan-sensitive control isolates. These included three mutations in *embC* (Thr270Ile, n = 2; Asn394, n = 2; Val981Leu, n = 1), one mutation in *embA* (Pro913Ser, n = 2), one mutation in *embB* (Glu378Ala, n = 2), and one mutation in *embR* (Cys110Tyr, n = 1). We considered non-synonymous mutations held by two or more ethambutol-resistant isolates for single nucleotide polymorphism (SNP) analysis. 

Some mutations occurred concurrently with other mutations in select isolates, and hence combinations of mutations are described here as mutation profiles in Table 2. In total, three mutation profiles were observed: (A) *embB*: Gly406Ala, (B) *embB*: Gly406Asp, and (C) *embC*: Leu333Arg, *embB*: Gly406Asp, and *embR*: Gln258fs. Profile A was seen in three isolates exhibiting resistance at ethambutol concentrations of 5 μg/mL (n = 2) and 3 μg/mL (n = 1). Profile B was observed in four isolates with resistance at 5 μg/mL (n = 2), 4 μg/mL (n = 1), and 3 μg/mL (n = 1) ethambutol. Profile C was observed in nine isolates and showed resistance at 4 μg/mL (n = 1), 3 μg/mL (n = 3), and 2 μg/mL (n = 5) ethambutol.

### 2.4. embB406 and Phenotypic Susceptibility to Additional First-Line Drugs

Phenotypic susceptibility to first-line drugs rifampin, isoniazid, and pyrazinamide was also assessed by critical concentration on the BACTEC™ MGIT™ 960 system (Table 2). In total, 93.8% (n = 15/16) isolates exhibiting an *embB* mutation were resistant to at least one additional first-line drug. Six isolates were resistant to rifampin, 15 were resistant to isoniazid, and two were resistant to pyrazinamide. All isolates with rifampin resistance were also resistant to isoniazid (n = 6), and were classified as multi-drug resistant (MDR). Two of these isolates were resistant to all three drugs: rifampin, isoniazid, and pyrazinamide. Both MDR isolates with additional resistance to pyrazinamide harboured an *embB* Gly406Asp mutation (n = 2). MDR isolates without pyrazinamide resistance held either a Gly406Asp mutation (n = 2) or Gly406Ala mutation (n = 2). Nine isolates were resistant to isoniazid only and all featured mutations in *embC* Leu333Arg, *embB* Gly406Asp, and *embR* Gln258fs. A single isolate was mono-resistant to ethambutol and had an *embB* Gly406Ala mutation.

## 3. Discussion

Between years 2002–2022, 2794 *M. tuberculosis* isolates were screened for *embB* mutations in our laboratory. In total, ethambutol resistance was observed in 3.08% (n = 86/2794) isolates included in this study. A previous report on Canadian resistance to anti-tuberculosis drugs in 2021 recorded ethambutol resistance in 0.72% of *M. tuberculosis* isolates tested for anti-tuberculosis drug resistance (n = 11/1536) [24]. The disparity between nationally reported rates for ethambutol resistance and our observations spanning 2002–2022 is likely due to the limitations of the culture collection included in this study. The NRCM does not receive all Canadian *Mycobacterium tuberculosis* complex (MTBC) isolates for DST, but rather receives MTBC from submitting Canadian laboratories with limited capacity for DST. As a result, this collection may not provide a complete picture of antimicrobial resistance among *M. tuberculosis* isolates in Canada. Outside of Canada, the rate of ethambutol resistance is reported to range from 0.3% to 14%, consistent with the present findings [25,26]. 

In total, *embB* mutations were observed in 5.01% (n = 140/2794) of *M. tuberculosis* isolates screened in this study, of which only 45.71% (n = 64/140) were phenotypically resistant to ethambutol. Notably, *embB* mutations were absent in 25.58% (n = 22/86) of ethambutol-resistant isolates in the present study. Accordingly, we show that the detection of an *embB* mutation in *M. tuberculosis* isolates corresponded to a PPV of 45.7%, specificity of 97.2%, and sensitivity of 74.4% for ethambutol phenotypic DST. These metrics show that single *embB* mutations provided low-confidence predictions of ethambutol resistance for this dataset. Other studies similarly report that *embB* mutations predicted ethambutol phenotypes with 81.3% sensitivity and 86.8% specificity, corroborating the low sensitivity of molecular DST employing *embB* mutations [16]. 

The 2023 WHO catalogue of MTBC mutations details molecular markers associated with drug resistance and classifies *embB* mutations according to a confidence grading system as “associated with resistance”, “associated with interim resistance”, “not associated with resistance”, or “uncertain significance” [19,20]. In the present study, the most abundant *embB* mutations “associated with resistance” occurred in codons 306 (n = 75), 406 (n = 16), and 497 (n = 5). Interestingly, discordance was recorded for each mutation observed at these loci, not only codon 406. 

In total, 69.3% (n = 52/75) of the study isolates with a single mutation in *embB*306 were phenotypically resistant. By mutation, resistance was observed in 86.5% of isolates with a Met306Val mutation (n = 32/37), 52.8% of isolates with a Met306Ile mutation (n = 19/36), and 50% of isolates with a Met306Leu mutation (n = 1/2). In comparison, WHO reports the PPV for each mutation: resistance was observed in 82.6% of isolates with a Met306Val mutation (n = 3245/3930), 62.8% of isolates with a Met306Ile mutation (n = 1953/3112), and 74.4% of isolates with a Met306Leu mutation (n = 145/195) [19]. Mutations in *embB*306 are well-studied and recognized as important molecular markers of ethambutol resistance [27]. Allelic exchange studies have identified that single *embB*306 mutations cause an increase in ethambutol minimum inhibitory concentration (MIC) [28], however, there is some debate in the literature as to whether single *embB*306 mutations are sufficient to induce clinically significant ethambutol resistance [27,28,29]. Here, we report that 30.7% (n = 23/75) of isolates with single *embB*306 mutations exhibit phenotypic susceptibility to ethambutol which is corroborated by several other studies that have identified *embB*306 mutations among ethambutol-susceptible isolates [21,29,30]. These results and literature show a low to moderate capacity for *embB*306 mutations to predict ethambutol resistance using sequencing-based molecular DST. 

A single resistance-associated mutation was observed in *embB* codon 497 (Gln497Arg) and 75% (n = 3/4) of isolates with this mutation were phenotypically resistant to ethambutol. WHO reports the PPV for Gln497Arg in which resistance was observed in 81.7% of isolates with this mutation (n = 999/1223) [19]. Like *embB*306, allelic exchange experiments investigating *embB*497 suggest that these mutations may only make low-to-moderate contributions to ethambutol resistance [28]. Again, these results and literature show moderate confidence in the ability of *embB*497 mutations to predict ethambutol resistance.

Other *embB* mutations “associated with resistance” were observed in singular ethambutol-resistant isolates in this study: Tyr319Ser, Asp328Tyr, Asp354Ala. Mutations were also observed that are “not associated with resistance” or have “uncertain significance” for ethambutol resistance according to the 2023 WHO mutation catalogue [19]. One mutation, Gly378Ala, is “not associated with resistance” and was observed in 37 isolates, of which only a single isolate was ethambutol-resistant. Four mutations categorized with “uncertain significance”, including Ile226Val, Ala313Val, Ala386Ser and Gln497Pro were observed in four isolates, all of which were phenotypically sensitive to ethambutol. Finally, *embB* mutation Tyr333Asn is not documented in the WHO mutation catalogue and was observed a single ethambutol-sensitive isolate.

The present study specifically evaluates ethambutol resistance among *M. tuberculosis* isolates with *embB*406 mutations due to the accumulating reports of sensitivity among isolates with this mutation [21,22,23,31,32,33,34]. Indeed, only 25% (n = 4/16) of isolates with *embB*406 mutations in the NRCM culture collection tested as ethambutol-resistant at the current critical concentration using the BACTEC™ MGIT™ 960 system. By mutation, 15.4% (n = 2/13) of isolates with a Gly406Asp mutation were reported as resistant and 66.6% (n = 2/3) of isolates with a Gly406Ala mutation were resistant. These data correspond to a PPV of 15.4%, specificity of 99.6%, and sensitivity of 2.3% for Gly406Asp and a PPV of 66.6%, specificity of 97.0%, and sensitivity of 2.3% for Gly406Ala. In comparison, WHO reports a PPV of 62.2%, specificity of 99.4%, and sensitivity of 3.5% for Gly406Ala and PPV of 56.4%, specificity of 99.4%, and sensitivity of 2.9% for Gly406Asp [19]. Our results in a limited number of isolates show that *embB*406 mutations are a low-confidence predictor of ethambutol resistance, and are corroborated by the WHO’s reports of low sensitivity and predictive values for these mutations. 

Interestingly, DST performed at 2, 3, and 4 µg/mL showed that all susceptible isolates with *embB*406 mutations exhibited ethambutol resistance undetectable by the current critical concentration. Here, we define low-level ethambutol resistance as resistance observed below the 5 µg/mL critical concentration for ethambutol. Stratifying by mutation, 84.6% (n = 11/13) Gly406Asp isolates and 33.3% (n = 1/3) Gly406Ala isolates exhibited resistance below 5 µg/mL. Specifically, 15.4% (n = 2/13) Gly406Asp isolates were resistant at 4 µg/mL, 30.8% (n = 4/13) Gly406Asp isolates and 33.3% (n = 1/3) Gly406Ala isolates were resistant at 3 µg/mL, and 38.5% (n = 5/13) Gly406Asp isolates were resistant at 2 µg/mL. Accordingly, these results illustrate that Gly406Asp and Gly406Ala mutations appear to be associated with low-level resistance. 

Here, we investigated discordance between molecular methods and phenotypic DST for ethambutol on the BACTEC™ MGIT™ 960 system. This discordance is not isolated to the BACTEC™ MGIT™ 960 system but is also reported for other WHO- and Clinical and Laboratory Standards Institute (CLSI)-endorsed susceptibility testing methods [6,19,20] and are summarized in Table 3. DST by agar proportion on solid media employs different critical concentrations for ethambutol depending on media type: 2 µg/mL by Löwenstein-Jensen (LJ), 5 µg/mL by Middlebrook 7H10 agar, and 7.5 µg/mL by Middlebrook 7H11 agar [6]. Few studies describe concordance between *embB*406 mutations and phenotypic DST using the agar proportion method [31,33]. Several report *embB*406 mutations among both resistant and susceptible isolates by agar proportion on LJ media [16,23,32,34,35]. Park et al. (2012) describe moderate ethambutol resistance (0.88–4 µg/mL) among *M. tuberculosis* isolates with *embB*406 mutations [23]. Others employing agar proportion DST have suggested that ethambutol resistance is multigenic and that *embB*406 mutations alone are insufficient for the development of resistance [21,28].

The BACTEC™ 460TB and BACTEC™ MGIT™ 960 systems employ agar proportion-equivalent ethambutol critical concentrations of 2.5 µg/mL and 5 µg/mL, respectively [6,40,41]. The adaptation of critical concentrations from agar proportion methods to the BACTEC™ 460TB system and the BACTEC™ MGIT™ 960 system shortly thereafter, has been criticized for poor concordance and standardization [15,42]. In the present study, 18 isolates with *embB* mutations associated with resistance were tested as phenotypically resistant by BACTEC™ 460TB (100% concordance between molecular and phenotypic DST). In contrast, 45 out of 80 isolates harbouring an *embB* mutation associated with resistance tested as resistant on the BACTEC™ MGIT™ 960 system (56.3% concordance between molecular and phenotypic DST). This study also detected one isolate with a Gly406Ala *embB* mutation that tested as ethambutol-sensitive on the BACTEC™ 460TB but resistant on the BACTEC™ MGIT™ 960 system. Several others also describe ethambutol-susceptible isolates with *embB*406 mutations [15,16,21,36]. Bwalya et al. (2022) and Christianson et al. (2014) report low-level ethambutol resistance among isolates with *embB*406 mutations [15,22]. These observations are often accompanied by a recommendation that the BACTEC™ MGIT™ 960 critical concentration may be lowered to ensure that resistance at or near the 5 µg/mL critical concentration is detected [15]. 

Microdilution plates have sought to address gaps between phenotypic DST methods by assessing MICs rather than critical concentrations for ethambutol [42]. Studies employing the Sensititre™ Mycobacterium tuberculosis MYCOTBI AST plate also report *embB*406 mutations among isolates exhibiting resistance below the 5 µg/mL breakpoint for ethambutol [37,38,39]. Liu et al. (2022) explain that *embB*406 mutations elevated ethambutol MICs at sub-threshold levels [37]. Li et al. (2022) record *embB*406 variants with MICs ranging from 1–16 µg/mL [38].

Previous studies have suggested that common mechanisms of resistance exist between isoniazid and ethambutol given their shared drug target [43] and synergistic activity [44]. Accordingly, we briefly investigated the susceptibility of isolates with *embB*406 mutations to the remaining three first-line anti-tuberculosis drugs: isoniazid, rifampin, and pyrazinamide. Ethambutol mono-resistance, while rare in literature, was observed in a single isolate with an *embB* Gly406Ala mutation. Isoniazid resistance was observed in 93.75% (n = 15/16) of isolates harbouring a mutation in *embB*406. Similarly, another study reported simultaneous resistance to ethambutol and isoniazid in 85.18% of *M. tuberculosis* isolates that underwent phenotypic DST [45]. However, additional studies are required to solidify a clear mechanistic linkage between resistance to ethambutol and other antimicrobial agents. 

Expanding the scope of this examination, we investigated other genes within the *embCAB* operon, including the operon’s regulator *embR*. As a result, it was found that nine isolates exhibited identical mutations in *embC* (Leu333Arg) and *embR* (Gln258fs) which have not been previously described, in addition to the *embB* Gly406Asp mutation. All nine isolates with this mutation profile were sensitive at 5 µg/mL ethambutol but exhibited low-level resistance undetectable at the critical concentration. Specifically, 11.1% (n = 1/9) were resistant at 4 µg/mL, 33.3% (n = 3/9) were resistant at 3 µg/mL, and 55.6% (n = 5/9) were resistant at 2 µg/mL. Notably, the *embR* frameshift mutation occurs within the forkhead-associated (FHA) domain of the operon’s regulator. As the FHA domain is critical for phosphorylation and activation of *embR* [46], we predict that this novel and disruptive frameshift mutation may impact *embCAB* expression and cause variability in resistance. Further investigation of these mutations is required to isolate their impact on ethambutol resistance. 

While this study included all strains with molecular and phenotypic DST in the Canadian National Reference Centre for Mycobacteriology culture collection, the limited number of strains with *embB*406 mutations included in this study must be acknowledged. Even so, our results corroborate a wealth of studies calling for improved concordance between molecular and phenotypic DST for ethambutol. The results herein illustrate that *embB*406 mutations are low-confidence mutations associated with low-level ethambutol resistance. It is possible that mutations outside of *embB*, including *embR*, may promote variability in ethambutol resistance. Molecular DST is an invaluable tool to rapidly inform patient treatment regimens while gold culture methods remain time-consuming with long turnaround times. However, discordance between molecular and phenotypic methods must be acknowledged with caution. A greater understanding of the impact of *embB* mutations on ethambutol resistance as well as the clinical significance of low-level resistance is required to inform the improvement of DST.

## 4. Materials and Methods

### 4.1. Study Isolates

The culture collection housed within the NRCM spanning 2002–2022 (n = 2794) was screened for *M. tuberculosis* isolates containing *embB* mutations by routine sequencing of the *embB* gene by WGS and Sanger sequencing. Screening identified 16 isolates harbouring an *embB*406 mutation, for which WGS-based SNP analysis and extended phenotypic ethambutol susceptibility testing were performed (described below). Pan-sensitive isolates lacking *embB* mutations (n = 10) and *M. tuberculosis* strain H37Rv ATCC 27294 were included in SNP analysis and extended phenotypic ethambutol susceptibility testing as control isolates.

### 4.2. Sanger Sequencing and Data Analysis

The *embB* region containing codon 406 was amplified using PCR primers F1 5′-TGATATTCGGCTTCCTGCTC-3′ and R1 5′- TGCACACCCAGTGTGAATG-3′ (ThermoFisher Scientific, Waltham, MA, USA) designed using Primer3 [47] (version 0.4.0). Primers were utilized at 0.2 µM in Amplitaq Gold™ 360 Master Mix (Applied Biosystems, Waltham, MA, USA). PCR amplification programs consisted of a 10-min initial denaturation at 95 °C followed by 30 cycles (94 °C, 30 s; 60 °C, 30 s; 72 °C, 30 s) and a final extension at 72 °C for 7 min. PCR products were purified using PCRClean™ DX (Aline Biosciences, Woburn, MA, USA) according to the manufacturer’s instructions (version Rev.2.10). Sanger sequencing was performed on a 3730xl DNA Analyzer (Applied Biosystems, Waltham, MA, USA) utilizing the same primers as for amplification. Sequence data from primers were paired and trimmed in SeqMan Pro 15 (DNASTAR Navigator 15, version 15.0.0.160) before MUSCLE alignment [48] to the *M. tuberculosis* H37Rv *embB* reference sequence (NC_000962.3: 4246514-4249810) in Geneious [49] (version 11.0.12). 

### 4.3. Whole Genome Sequencing

DNA libraries were prepared with the Illumina^®^ DNA Prep kit (Illumina, San Diego, CA, USA) using a modified quarter-volume protocol before sequencing on the MiSeq platform with a MiSeq Reagent Kit v3 (500-cycle; Illumina, San Diego, CA, USA) to generate 2 × 250 bp paired-end reads. Completed sequencing runs were uploaded to the Integrated Rapid Infectious Disease Analysis (IRIDA) Platform [50]. Trimmomatic [51] in Galaxy (version 0.36.5) was utilized to trim sequences resulting from adaptor read-through (ILLUMINACLIP:Nextera-PE:2:30:10:8) and poor-quality reads (SLIDINGWINDOW:5:20). Read quality was assessed with FastQC [52] (version 0.72). Kraken2 [53] (version 2.2) was employed to detect potential contaminating microbial DNA. BioHansel [54] (version 1.2), an in-house developed tool, was utilized for *M. tuberculosis* complex differentiation. MyKrobe Predictor [18] (version 0.10.0) was utilized for genotypic DST for first- and second-line anti-tuberculosis drugs as part of routine diagnostic workflows. 

SNP analysis was performed for strains with *embB*406 mutations and pan-sensitive control strains. Using Snippy [55] (https://github.com/tseemann/snippy, accessed on 29 April 2024). sequencing reads were assembled and compared to an annotated *M. tuberculosis* H37Rv reference genome (AL_123456.3) for SNP calling. The quality of the alignment used for SNP detection was determined with QualiMap2 [56] (version 2.2.2d). Minimum quality thresholds for alignments included genome coverage of 30, 90% of reads mapping to the reference genome, a mapping quality of 58.5, and GC content of 65 ± 1%. Alignments were visualized in Geneious [49] (version 11.0.12). SNVPhyl [57] (version 1.2.3) was used for the phylogenomic analysis of all strains in the study, utilizing the *M. tuberculosis* H37Rv genome as a reference (NC_000962.3). Microreact [58] (https://microreact.org, accessed on 29 April 2024) was used to visualize phylogenic trees.

### 4.4. Phenotypic Drug Susceptibility Testing

DST for ethambutol was performed on the radiometric BACTEC™ 460TB system (Becton, Dickinson and Company, Sparks, MD, USA) from 2002 to mid-2007. Following, routine DST was performed on the fluorometric BD BACTEC™ MGIT™ 960 automated mycobacterial detection system (Becton, Dickinson and Company, Franklin Lakes, NJ, USA) with the BD BBL™ MGIT™ AST SIRE kit (Becton, Dickinson and Company, Franklin Lakes, NJ, USA). All isolates were tested at the critical concentration for ethambutol as recommended by the manufacturer and CLSI: 2.5 µg/mL on the BACTEC™ 460TB system and 5.0 µg/mL on the MGIT™ 960 system [6]. 

Extended DST was performed for strains containing *embB*406 mutations and pan-sensitive control isolates, in which susceptibility to ethambutol concentrations of 4 μg/mL, 3 μg/mL, and 2 μg/mL was tested on the MGIT™ 960 system. In addition, DST for the remaining three first-line anti-tuberculosis drugs was tested on the MGIT™ 960 system at the following critical concentrations according to manufacturer and CLSI recommendations: 1.00 µg/mL rifampin, 0.1 µg/mL isoniazid, and 100 µg/mL pyrazinamide [6]. DST for all isolates was performed in duplicate and in parallel with *M. tuberculosis* H37Rv ATCC27294 control strain. 

## Figures and Tables

**Table 1:** List of *embB* Mutations and Corresponding Phenotypic Susceptibility Testing Data for Study Isolates from Years 2002–2022.

*embB* Mutation	Phenotype ^a^	Number of Isolates Tested by MGIT960 ^b^ (5 µg/mL)	Number of Isolates Tested by BACTEC460 ^c^ (2.5 µg/mL)	Number of Isolates	WHO Confidence Grading of Resistance Mutations ^d^
Met306Val	S	5	0	5	“Associated with Resistance”
	R	22	10	32	
Met306Ile	S	17	0	17	
	R	16	3	19	
Met306Leu	S	1	0	1	
	R	0	1	1	
Tyr319Ser	S	0	0	0	
	R	0	1	1	
Asp328Tyr	S	0	0	0	
	R	1	0	1	
Asp354Ala	S	0	0	0	
	R	1	0	1	
**Gly406Ala ^e^**	S	**0**	**0**	**0**	
	R	**1**	**2**	**3**	
**Gly406Asp**	S	**11**	**0**	**11**	
	R	**1**	**1**	**2**	
Gln497Arg	S	1	0	1	
	R	3	0	3	
Glu378Ala	S	36	0	36	“Not Associated with Resistance”
	R	1	0	1	
Ile226Val	S	1	0	1	“Uncertain Significance”
	R	0	0	0	
Ala313Val	S	1	0	1	
	R	0	0	0	
Ala386Ser	S	1	0	1	
	R	0	0	0	
Gln497Pro	S	1	0	1	
	R	0	0	0	
Tyr333Asn	S	1	0	1	Not Documented in WHO Catalogue
	R	0	0	0	
Wild-type	S	2593	39	2632	Not applicable
	R	8	14	22	

Bold text indicates *embB*406 mutations. ^a^ S: sensitive; R: resistant. ^b^ MGIT960: BACTEC™ MGIT™ 960 system. ^c^ BACTEC460: BACTEC™ 460TB system. ^d^ Significance according to the 2023 WHO catalogue of mutations in *M. tuberculosis* complex and their association with drug resistance or susceptibility [19]. ^e^ One isolate reported as resistant by routine phenotypic testing on the BACTEC™ 460TB system exhibited a sensitive result when subsequently tested on the BACTEC™ MGIT™ 960 system for confirmation of DST result.

**Table 2:** A Summary of Drug Resistance and *embCAB* Mutation Profiles of *M. tuberculosis* Isolates.

Isolate Number	Mutation Profile				Phenotypic Drug Susceptibility Testing Results ^a^				
	*emb* *C*	*emb* *A*	*emb* *B*	*emb* *R*	EMB		RIF	INH	PZA
					Highest Concentration of Resistance (μg/mL)	Phenotype			
3	-	-	Gly406Ala	-	5	R	S	S	S
15					5	R	R	R	S
2					3	S	R	R	S
1	-	-	Gly406Asp	-	5	R	R	R	R
11					5	R	R	R	S
4					4	S	R	R	R
5					3	S	R	R	S
6	Leu333Arg	-	Gly406Asp	Gln258fs	4	S	S	R	S
7					3	S	S	R	S
10					3	S	S	R	S
12					3	S	S	R	S
8					2	S	S	R	S
9					2	S	S	R	S
13					2	S	S	R	S
14					2	S	S	R	S
16					2	S	S	R	S

^a^ First-line drug sensitivity phenotype interpreted at the critical concentration: ethambutol (EMB) at 5 µg/mL; rifampin (RIF) at 1 µg/mL; isoniazid (INH) at 0.1 µg/mL; pyrazinamide (PZA) at 100 µg/mL. R: resistant; S: susceptible. fs: frameshift.

**Table 3:** Ethambutol DST and *embB*406 Mutations among Literature.

Study	Gly406Asp		Gly406Ala		Method, Critical Concentration
	Susceptible	Resistant	Susceptible	Resistant	
Plinke et al., 2010 [31]	0	5	0	2	LJ ^a^, 2 µg/mL
Park et al., 2012 [23]	4	2	2	1	LJ ^a^, 2 µg/mL
Zhao et al., 2015 [35]	3	2	2	4	LJ ^a^, 2 µg/mL
Brossier et al., 2015 [33]	0	4	0	2	LJ ^a^, 2 µg/mL
Xu et al., 2015 [34]	2	2	2	5	LJ ^a^, 2 µg/mL
Tylyaprawat et al., 2019 [32]	4	1	0	2	Agar proportion, 5 µg/mL
Bwalya et al., 2022 [22]	3	0	2	1	M960 ^b^
Al Mahrouqi 2022 [36]	2	0	0	0	M960 ^b^
Li et al., 2020 [16]	2	0	0	3	LJ, 2 ug/mL; M960, MABA ^c^
Liu et al., 2022 [37]	6	5	21	7	MYCOTBI, MIC ^d^
Li et al., 2022 [38]	3	0	2	8	MYCOTBI, MIC ^d^
Finci et al., 2022 [39]	4	0	1	0	MYCOTBI, MIC ^d^
Total	31	21	32	32	
WHO [19]	212	274	199	328	All methods ^e^

^a^ LJ, Löwenstein-Jensen media. ^b^ BACTEC™ MGIT™ 960 system. ^c^ MABA, Microplate Alamar Blue assay. ^d^ MIC interpreted with a 5 µg/mL ethambutol breakpoint. ^e^ Phenotypic DST performed using all methods approved by the WHO [19].

## Data Availability

All datasets generated and analyzed during the current study are available in the NCBI Sequence Read Archive under BioProject ID PRJNA928676, https://www.ncbi.nlm.nih.gov/bioproject/PRJNA928676/ (accessed on 2 July 2024).

## References

[1] World Health Organization Global Tuberculosis Report 2022. https://www.who.int/teams/global-tuberculosis-programme/tb-reports/global-tuberculosis-report-2022.

[2] Schön T., Miotto P., Köser C.U., Viveiros M., Böttger E., Cambau E. (2017). *Mycobacterium tuberculosis* drug-resistance testing: Challenges, recent developments and perspectives. Clin. Microbiol. Infect..

[3] Long R., Avendano M., Kunimoto D. (2014). Chapter 8: Drug-Resistant Tuberculosis. Canadian Tuberculosis Standards.

[4] Brode S.K., Dwilow R., Kunimoto D., Menzies D., Khan F.A. (2022). Chapter 8: Drug-resistant tuberculosis. Can. J. Respir. Crit. Care Sleep Med..

[5] Public Health Agency of Canada, Tuberculosis in Canada: Infographic (2022). https://www.canada.ca/en/public-health/services/publications/diseases-conditions/tuberculosis-canada-infographic-2022.html.

[6] Woods G.L., Brown-Elliott B.A., Conville P.S., Desmond E.P., Hall G.S., Lin G., Pfyffer G.E., Ridderhof J.C., Siddiqi S.H., Wallace R.J. (2018). Susceptibility Testing of Mycobacteria, Nocardiae, and Other Aerobic Actinomycetes.

[7] Wilson M., DeRisi J., Kristensen H.H., Imboden P., Rane S., Brown P.O., Schoolnik G.K. (1999). Exploring drug-induced alterations in gene expression in *Mycobacterium tuberculosis* by microarray hybridization. Proc. Natl. Acad. Sci. USA.

[8] Spinato J., Boivin É., Bélanger-Trudelle É., Fauchon H., Tremblay C., Soualhine H. (2016). Genotypic characterization of drug resistant *Mycobacterium tuberculosis* in Quebec, 2002–2012. BMC Microbiol..

[9] Bolotin S., Alexander D.C., Chedore P., Drews S.J., Jamieson F. (2009). Molecular characterization of drug-resistant *Mycobacterium tuberculosis* isolates from Ontario, Canada. J. Antimicrob. Chemother..

[10] Kohli M., Schiller I., Dendukuri N., Dheda K., Denkinger C.M., Schumacher S.G., Steingart K.R. (2018). Xpert^®^ MTB/RIF assay for extrapulmonary tuberculosis and rifampicin resistance. Cochrane Database Syst. Rev..

[11] Pankhurst L.J., del Ojo Elias C., Votintseva A.A., Walker T.M., Cole K., Davies J., Fermont J.M., Gascoyne-Binzi D.M., Kohl T.A., Kong C. (2016). Rapid, comprehensive, and affordable mycobacterial diagnosis with whole-genome sequencing: A prospective study. Lancet Respir. Med..

[12] Gygli S.M., Keller P.M., Ballif M., Blöchliger N., Hömke R., Reinhard M., Loiseau C., Ritter C., Sander P., Borrell S. (2019). Whole-Genome Sequencing for Drug Resistance Profile Prediction in *Mycobacterium tuberculosis*. Antimicrob. Agents Chemother..

[13] Walker T.M., Kohl T.A., Omar S.V., Hedge J., Elias C.D.O., Bradley P., Iqbal Z., Feuerriegel S., Niehaus K.E., Wilson D.J. (2015). Modernizing Medical Microbiology (MMM) Informatics Group. Whole-genome sequencing for prediction of *Mycobacterium tuberculosis* drug susceptibility and resistance: A retrospective cohort study. Lancet Infect. Dis..

[14] Krüüner A., Yates M.D., Drobniewski F.A. (2006). Evaluation of MGIT 960-Based Antimicrobial Testing and Determination of Critical Concentrations of First-and Second-Line Antimicrobial Drugs with Drug-Resistant Clinical Strains of *Mycobacterium tuberculosis*. J. Clin. Microbiol..

[15] Christianson S., Voth D., Wolfe J., Sharma M.K. (2014). Re-Evaluation of the Critical Concentration for Ethambutol Antimicrobial Sensitivity Testing on the MGIT 960. PLoS ONE.

[16] Li M., Chen R., Lin S., Lu Y., Liu H., Li G., Liu Z., Zhao X., Zhao L., Wan K.-L. (2020). Detecting Ethambutol Resistance in *Mycobacterium tuberculosis* Isolates in China: A Comparison Between Phenotypic Drug Susceptibility Testing Methods and DNA Sequencing of *embAB*. Front. Microbiol..

[17] Miotto P., Zhang Y., Cirillo D.M., Yam W.C. (2018). Drug resistance mechanisms and drug susceptibility testing for tuberculosis. Respirology.

[18] Bradley P., Gordon N.C., Walker T.M., Dunn L., Heys S., Huang B., Earle S., Pankhurst L.J., Anson L., De Cesare M. (2015). Rapid antibiotic-resistance predictions from genome sequence data for *Staphylococcus aureus* and *Mycobacterium tuberculosis*. Nat. Commun..

[19] World Health Organization Catalogue of Mutations in Mycobacterium tuberculosis Complex and Their Association with Drug Resistance, Second Edition. https://www.who.int/publications/i/item/9789240082410.

[20] Walker T.M., Miotto P., Köser C.U., Fowler P.W., Knaggs J., Iqbal Z., Hunt M., Chindelevitch L., Farhat M.R., Cirillo D.M. (2022). The CRyPTIC Consortium; the Seq&Treat Consortium. The 2021 WHO catalogue of *Mycobacterium tuberculosis* complex mutations associated with drug resistance: A genotypic analysis. Lancet Microbe.

[21] Shi D., Li L., Zhao Y., Jia Q., Li H., Coulter C., Jin Q., Zhu G. (2011). Characteristics of *embB* mutations in multidrug-resistant *Mycobacterium tuberculosis* isolates in Henan, China. J. Antimicrob. Chemother..

[22] Bwalya P., Solo E.S., Chizimu J.Y., Shrestha D., Mbulo G., Thapa J., Nakajima C., Suzuki Y. (2022). Characterization of *embB* mutations involved in ethambutol resistance in multi-drug resistant *Mycobacterium tuberculosis* isolates in Zambia. Tuberculosis.

[23] Park Y.K., Ryoo S.W., Lee S.H., Jnawali N., Kim C.-K., Kim H.J., Kim S.J. (2012). Correlation of the phenotypic ethambutol susceptibility of *Mycobacterium tuberculosis* with *embB* gene mutations in Korea. J. Med. Microbiol..

[24] Public Health Agency of Canada Tuberculosis Surveillance in Canada Summary Report: 2012–2021. https://www.canada.ca/en/public-health/services/publications/diseases-conditions/tuberculosis-surveillance-canada-summary-2012-2021.html.

[25] Seyoum B., Demissie M., Worku A., Bekele S., Aseffa A. (2014). Prevalence and Drug Resistance Patterns of *Mycobacterium tuberculosis* among New Smear Positive Pulmonary Tuberculosis Patients in Eastern Ethiopia. Tuberc. Res. Treat..

[26] Nasiri M.J., Imani Fooladi A.A., Dabiri H., Pormohammad A., Salimi Chirani A., Dadashi M., Houri H., Heidary M., Feizabadi M.M. (2016). Primary ethambutol resistance among Iranian pulmonary tuberculosis patients: A systematic review. Ther. Adv. Infect. Dis..

[27] Starks A.M., Gumusboga A., Plikaytis B.B., Shinnick T.M., Posey J.E. (2009). Mutations at *embB* Codon 306 Are an Important Molecular Indicator of Ethambutol Resistance in *Mycobacterium tuberculosis*. Antimicrob. Agents Chemother..

[28] Safi H., Fleischmann R.D., Peterson S.N., Jones M.B., Jarrahi B., Alland D. (2010). Allelic Exchange and Mutant Selection Demonstrate that Common Clinical *embCAB* Gene Mutations Only Modestly Increase Resistance to Ethambutol in *Mycobacterium tuberculosis*. Antimicrob. Agents Chemother..

[29] Safi H., Sayers B., Hazbón M.H., Alland D. (2008). Transfer of *embB* Codon 306 Mutations into Clinical *Mycobacterium tuberculosis* Strains Alters Susceptibility to Ethambutol, Isoniazid, and Rifampin. Antimicrob. Agents Chemother..

[30] Hernando Hazbón M., Bobadilla del Valle M., Inírida Guerrero M., Varma-Basil M., Filliol I., Cavatore M., Colangeli R., Safi H., Billman-Jacobe H., Lavender C. (2005). Role of *embB* Codon 306 Mutations in *Mycobacterium tuberculosis* Revisited: A Novel Association with Broad Drug Resistance and IS6110 Clustering Rather than Ethambutol Resistance. Antimicrob. Agents Chemother..

[31] Plinke C., Cox H.S., Zarkua N., Karimovich H.A., Braker K., Diel R., Rü sch-Gerdes S., Feuerriegel S., Niemann S. (2010). *embCAB* sequence variation among ethambutol-resistant *Mycobacterium tuberculosis* isolates without *embB*306 mutation. J. Antimicrob. Chemother..

[32] Tylyaprawat O., Chaiprasert A., Chongtrakool P. (2019). Distribution of *embB* mutations of Thai clinical isolates of ethambutol-resistant *Mycobacterium tuberculosis*. J. Glob. Antimicrob. Resist..

[33] Brossier F., Sougakoff W., Bernard C., Petrou M., Adeyema K., Pham A., De La Breteque D.A., Vallet M., Jarlier V., Sola C. (2015). Molecular Analysis of the *embCAB* Locus and *embR* Gene Involved in Ethambutol Resistance in Clinical Isolates of *Mycobacterium tuberculosis* in France. Antimicrob. Agents Chemother..

[34] Xu Y., Jia H., Huang H., Sun Z., Zhang Z. (2015). Mutations Found in *embCAB*, *embR*, and *ubiA* Genes of Ethambutol-Sensitive and -Resistant *Mycobacterium tuberculosis* Clinical Isolates from China. Biomed. Res. Int..

[35] Zhao L., Sun Q., Liu H., Wu X., Xiao T., Zhao X., Li G., Jiang Y., Zeng C., Wan K. (2015). Analysis of *embCAB* Mutations Associated with Ethambutol Resistance in Multidrug-Resistant *Mycobacterium tuberculosis* Isolates from China. Antimicrob. Agents Chemother..

[36] Al Mahrouqi S., Gadalla A., Azri S., Al-Hamidi S., Al-Jardani A., Balkhair A., Al-Fahdi A., Balushi L., Zadjali S., Al Marhoubi A. (2022). Drug Resistant *Mycobacterium tuberculosis* in Oman: Resistance-conferring mutations and lineage diversity. PeerJ.

[37] Liu D., Huang F., Zhang G., He W., Ou X., He P., Zhao B., Zhu B., Liu F., Li Z. (2022). Whole-genome sequencing for surveillance of tuberculosis drug resistance and determination of resistance level in China. Clin. Microbiol. Infect..

[38] Li J., Yang T., Hong C., Yang Z., Wu L., Gao Q., Yang H., Tan W., Paula D.A., Carvalho-Assef A. (2022). Whole-Genome Sequencing for Resistance Level Prediction in Multidrug-Resistant Tuberculosis. Microbiol. Spectr..

[39] Finci I., Merker M., Barilar I., Kohl T.A., Niemann S., Andres S., Kranzer K., Maurer F.P., Albertini S.A., Hoogland C. (2022). Investigating resistance in clinical *Mycobacterium tuberculosis* complex isolates with genomic and phenotypic antimicrobial susceptibility testing: A multicentre observational study. Lancet Microbe.

[40] Siddiqi S.H., Rusch-Gerdes S. (2006). BACTEC MGIT 960 TB System Product and Procedure Manual.

[41] Siddiqi S.H. (1995). BACTEC 460 TB System Product and Procedure Manual.

[42] Lee J., Armstrong D.T., Ssengooba W., Park J.-A., Yu Y., Mumbowa F., Namaganda C., Mboowa G., Nakayita G., Armakovitch S. (2014). Sensititre MYCOTB MIC Plate for Testing *Mycobacterium tuberculosis* Susceptibility to First-and Second-Line Drugs. Antimicrob. Agents Chemother..

[43] Palomino J.C., Martin A. (2014). Drug resistance mechanisms in *Mycobacterium tuberculosis*. Antibiotics.

[44] Zhu C., Liu Y., Hu L., Yang M., He Z.-G. (2018). Molecular mechanism of the synergistic activity of ethambutol and isoniazid against *Mycobacterium tuberculosis*. J. Biol. Chem..

[45] Gupta P., Jadaun G., Das R., Gupta U., Srivastava K., Chauhan A., Sharma V., Chauhan D., Katoch V. (2006). Simultaneous ethambutol and isoniazid resistance in clinical isolates of *Mycobacterium tuberculosis*. Indian. J. Med. Res..

[46] Molle V., Kremer L., Girard-Blanc C., Besra G.S., Cozzone A.J., Prost J.-F. (2003). An FHA Phosphoprotein Recognition Domain Mediates Protein EmbR Phosphorylation by PknH; a Ser/Thr Protein Kinase from *Mycobacterium tuberculosis*. Biochemistry.

[47] Untergasser A., Cutcutache I., Koressaar T., Ye J., Faircloth B., Remm M., Rozen S. (2012). Primer3--new capabilities and interfaces. Nucleic Acids Res..

[48] Edgar R. (2004). MUSCLE: Multiple sequence alignment with high accuracy and high throughput. Nucleic Acids Res..

[49] Geneious Prime Version 2022.11.0.12. https://www.geneious.com.

[50] Matthews T.C., Bristow F.R., Griffiths E.J., Petkau A., Adam J., Dooley D., Kruczkiewicz P., Curatcha J., Cabral J., Fornika D. (2018). The Integrated Rapid Infectious Disease Analysis (IRIDA) Platform. bioRxiv.

[51] Bolger A.M., Lohse M., Usadel B. (2014). Genome analysis Trimmomatic: A flexible trimmer for Illumina sequence data. Bioinformatics.

[52] FastQC Version 0.72. https://www.bioinformatics.babraham.ac.uk/projects/fastqc/.

[53] Wood D.E., Lu J., Langmead B. (2019). Improved metagenomic analysis with Kraken 2. Genome Biol..

[54] Labbé G., Kruczkiewicz P., Robertson J., Mabon P., Schonfeld J., Kein D., Rankin M.A., Gopez M., Hole D., Son D. (2021). Rapid and accurate SNP genotyping of clonal bacterial pathogens with BioHansel. Microb. Genom..

[55] Snippy. https://github.com/tseemann/snippy.

[56] Okonechnikov K., Conesa A., García-Alcalde F. (2016). Qualimap 2: Advanced multi-sample quality control for high-throughput sequencing data. Bioinformatics.

[57] Petkau A., Mabon P., Sieffert C., Knox N.C., Cabral J., Iskander M., Iskander M., Weedmark K., Zaheer R., Katz L.S. (2017). SNVPhyl: A single nucleotide variant phylogenomics pipeline for microbial genomic epidemiology. Microb. Genom..

[58] Microreact. https://microreact.org/.

